# Supplementary material for: Loneliness and Increased Hazardous Alcohol Use: Data from a Nationwide Internet Survey with 1-Year Follow-Up
Source: Int J Environ Res Public Health. 2022 Sep 24;19(19):12086. doi: 10.3390/ijerph191912086 (PMC9566210; doi:10.3390/ijerph191912086)
Supplement: Supplementary file 1 [file ijerph-19-12086-s001.zip › ijerph-1917836-supplementary.pdf]

Supplementary Figure S1. The flow diagram of the study population

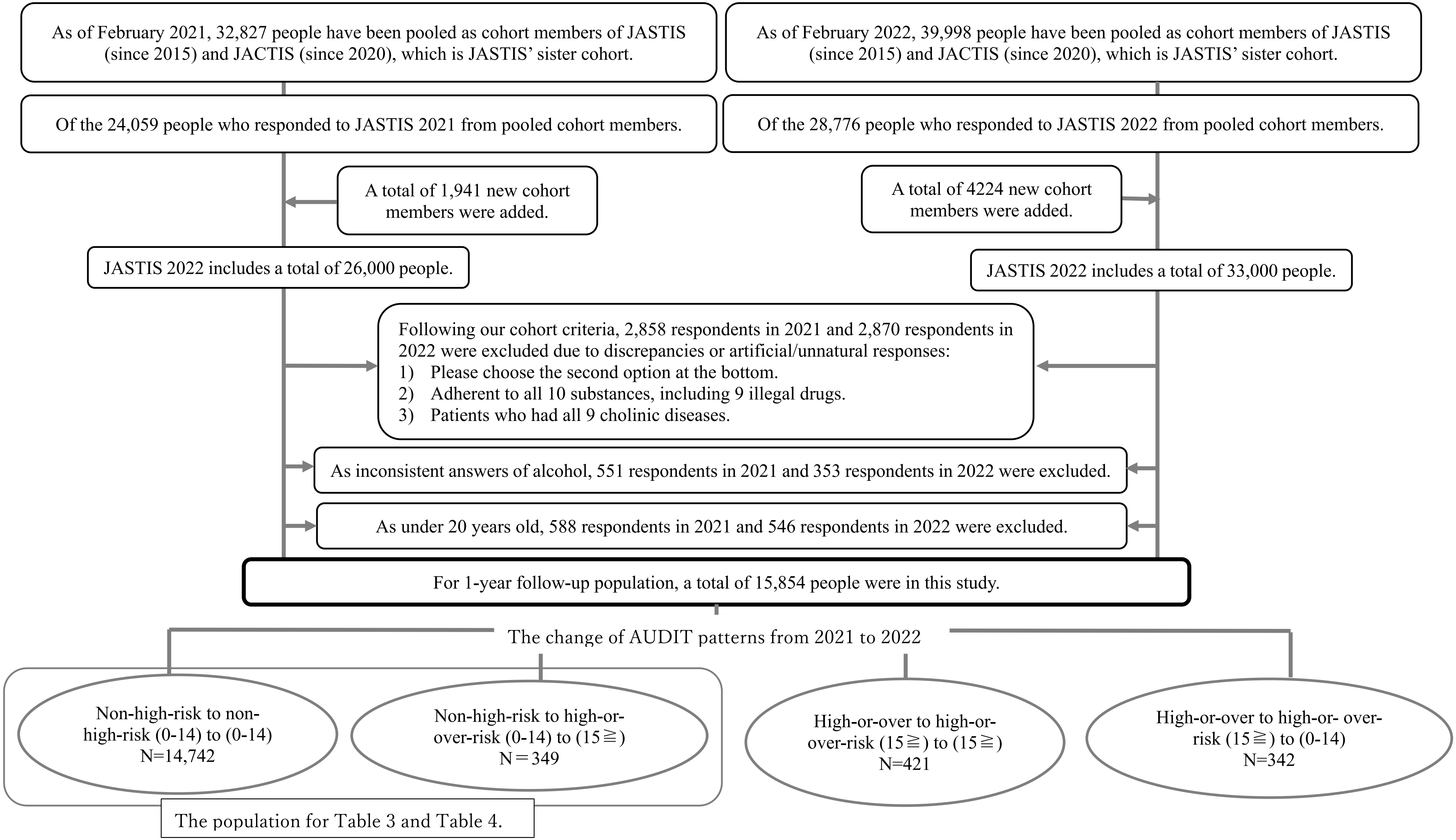

Caption

Figure S1. The flow diagram of the study population

The figure shows the steps used in the selection of the members of this study. Information on the two cohort studies is available on the website: JASTIS (https://jastis-study.jp/), and JACSIS (https://jacsis-study.jp/index.html).

Supplementary Table S1. AUDIT questions and each score of answers

	0	1	2	3	4
1. How often do you have a drink containing alcohol?	Never	Monthly or less	2 to 4 times a month	2 to 3 times a week	4 or more times a week
2. How many drinks containing alcohol do you have on a typical day when you are drinking?	1 or 2	3 or 4	5 or 6	7, 8, or 9	10 or more
3. How often do you have six or more drinks on one occasion?	Never	Less than monthly	Monthly	Weekly	Daily or almost daily
4. How often during the last year have you found that you were not able to stop drinking once you had started?	Never	Less than monthly	Monthly	Weekly	Daily or almost daily
5. How often during the last year have you failed to do what was normally expected from you because of drinking?	Never	Less than monthly	Monthly	Weekly	Daily or almost daily
6. How often during the last year have you needed a first drink in the morning to get yourself going after a heavy drinking session?	Never	Less than monthly	Monthly	Weekly	Daily or almost daily
7. How often during the last year have you had a feeling of guilt or remorse after drinking?	Never	Less than monthly	Monthly	Weekly	Daily or almost daily
8. How often during the last year have you been unable to remember what happened the night before because you had been drinking?	Never	Less than monthly	Monthly	Weekly	Daily or almost daily
9. Have you or someone else been injured as a result of your drinking?	No		Yes, but not in the last year		Yes, during the last year
10. Has a relative or friend or a doctor or another health worker been concerned about your drinking or suggested you cut down?	No		Yes, but not in the last year		Yes, during the last year

Supplementary Table S2. The scale score of our study based on the 3-item of the University of California, Los Angeles Loneliness Scale version 3, UCLA-LS3 (SF-3), and the original score scale on JASTIS questionnaire

<table>
<tr><td rowspan="2"></td><td colspan="5">The scale score in our study based on UCLA-LS3 (SF-3)</td></tr>
<tr><td colspan="2">Hardly ever</td><td>Some of the time</td><td colspan="2">Often</td></tr>
<tr><td>1. How often do you feel that you lack companionship?</td><td colspan="2">1</td><td>2</td><td colspan="2">3</td></tr>
<tr><td>2. How often do you feel left out?</td><td colspan="2">1</td><td>2</td><td colspan="2">3</td></tr>
<tr><td>3. How often do you feel isolated from others?</td><td colspan="2">1</td><td>2</td><td colspan="2">3</td></tr>
<tr><td rowspan="2">The original score scale on JASTIS 2021</td><td>1</td><td>2</td><td>3</td><td>4</td><td>5</td></tr>
<tr><td>Never</td><td>Rarely</td><td>Sometime</td><td>Usually</td><td>Always</td></tr>
</table>

Supplementary Table S3. The four patterns of AUDIT categories' changes and their characteristics based on 2021
(The two patterns were shown in Table 3.)

	The change of AUDIT patterns from 2021 to 2022 (N=15,854)			
	Non-high-risk to non-high-risk (0-14) to (0-14)	Non-high-risk to high or over (0-14) to (15≧)	Over-high-risk to high or over (15≧)to (15≧)	High or over to non-high-risk (15≧)to (0-14)
	N＝14,742	N＝349	N＝421	N＝342
	N （%）	N （%）	N （%）	N （%）
Loneliness(Mean, SD）	4.0(±1.8）	4.4(±1.9）	5.0(±2.2）	4.7(±2.1）
Score<6	11,918(81)	254(73)	258(61)	218(64)
Score≧6	2,824(19)	95(27)	163(39)	124(36)
Age, year (Mean, SD)	53.0(±15.7）	49.9(±14.5)	49.2(±15.0)	46.9(±16.4)
20－39 years	3,246(22)	91(26)	85(20)	91(27)
40－59 years	5,695(39)	160(46)	235(56)	156(46)
60－79 years	5,801(39)	98(28)	101(24)	95(28)
Sex				
Women	7,382(50)	67(19)	89(21)	77(23)
Men	7,360(50)	282(81)	332(79)	265(77)
Education				
Low	4,561(31)	86(25)	147(35)	105(31)
Middle	3,173(22)	71(20)	53(13)	45(13)
High	7,008(48)	192(55)	221(52)	192(56)
Marital status				
Marriage	9,398(64)	233(67)	272(65)	218(64)
No marriage	3,810(26)	83(24)	99(24)	93(27)
Divorced/Widowed	1,534(10)	33(9)	50(12)	31(9)
Living arrangements				
Living with someone	11,987(81)	274(79)	325(77)	263(77)
Living alone	2,755(19)	75(21)	96(23)	79(23)
Job				
Regular job	6,093(41)	215(62)	278(66)	237(69)
No-regular employee	2,800(19)	56(16)	64(15)	44(13)
No main income job as students /retiree/ housework	3,716(25)	37(11)	24(6)	21(6)
Unemployed	2,133(14)	41(12)	55(13)	40(12)
Income				
Under 2 million yen	2,366(16)	51(15)	65(15)	55(16)
2-4 million yen	5,354(36)	137(39)	158(38)	120(35)
4-6 million yen	2,342(16)	61(17)	83(20)	64(19)
6-10 million yen	1,477(10)	55(16)	66(16)	52(15)
10 million or more	327(2)	7(2)	19(5)	14(4)
Don't know/want to answer	2,876(20)	38(11)	30(7)	37(11)
Current smoker				
No	12,075(82)	230(66)	250(59)	208(61)
Yes	2,667(18)	119(34)	171(41)	134(39)
Phycological distress (Mean, SD）	4.4(±5.4）	6.0(±6.2）	7.3(±6.7）	7.3(±6.5）
K6 ＜13	13,370(91)	299(86)	331(79)	268(78)
K6 ≧13	1,372(9)	50(14)	90(21)	74(22)

Supplementary Table S4. The percentage and means of loneliness score in each AUDIT item among follow-up populations

	AUDIT items†	Loneliness (≧6) N=3,949	Loneliness score N=15,854	
	Harmful alcohol use	**n (%)**	**Mean (SD)**	Bonferroni test*
Q1	Frequency of drinking			
	0 (Non-drinker)	1050(33)	4.16(±1.89)	0>3, 0>4
	1 (Monthly or less)	658(21)	4.19(±1.91)	1>3, 1>4
	2 (2-4 times a month)	461(14)	4.10(±1.88)	
	3 (2-3 times a week)	379(12)	4.02(±1.77)	3>4
	4 (4 or more times a week)	658(21)	3.86(±1.66)	
Q2	Typical quantity on a day			
	0 (1 or 2)	1267(40)	3.99(±1.78)	
	1 (3 or 4)	502(16)	4.02(±1.78)	
	2 (5 or 6)	210(7)	4.01(±1.75)	
	3 (7 or 9)	66(2)	4.02(±1.80)	
	4 (10 or more)	111(3)	4.43(±2.14)	4>0, 4>2, 4>3
Q3	Frequency of heavy drinking			
	0 (Never)	1363(43)	3.97(±1.76)	
	1 (Less than monthly)	339(11)	4.06(±1.83)	
	2 (Monthly)	144(4)	4.25(±1.86)	2>0
	3 (Weekly)	155(5)	4.14(±1.89)	
	4 (Daily or almost daily)	155(5)	4.09(±1.88)	
	Sympotom of disease			
Q4	Impaired control over drinking			
	0 (Never)	1772(55)	3.94(±1.73)	
	1 (Less than monthly)	165(5)	4.55(±2.07)	1>0
	2 (Monthly)	105(3)	4.98(±2.08)	2>0, 2>1
	3 (Weekly)	61(2)	5.13(±2.24)	3>0, 3>1
	4 (Daily or almost daily)	53(2)	4.76(±2.29)	4>0
Q5	Increased fail to do because of drinking			
	0 (Never)	1772(55)	3.94(±1.73)	
	1 (Less than monthly)	165(5)	4.53(±2.11)	1>0
	2 (Monthly)	105(3)	5.07(±2.09)	2>0, 2>1
	3 (Weekly)	61(2)	4.85(±2.21)	3>0
	4 (Daily or almost daily)	53(2)	4.92(±2.37)	4>0
Q6	Morning drinking			
	0 (Never)	1912(60)	3.96(±1.75)	
	1 (Less than monthly)	86(3)	4.93(±2.25)	1>0
	2 (Monthly)	79(2)	5.63(±2.04)	2>0, 2>1, 2>4

	3 (Weekly)	41(1)	5.30(±2.33)	3>0
	4 (Daily or almost daily)	38(1)	4.86(±2.30)	4>0
	Harmful alcohol use			
Q7	Guilt after drinking			
	0 (Never)	1730(54)	3.92(±1.72)	
	1 (Less than monthly)	183(6)	4.54(±2.04)	1>0
	2 (Monthly)	107(3)	5.20(±2.20)	2>0, 2>1
	3 (Weekly)	82(3)	5.70(±2.28)	3>0, 3>1
	4 (Daily or almost daily)	54(2)	5.27(±2.38)	4>0, 4>1
Q8	Blackouts			
	0 (Never)	1757(55)	3.95(±1.75)	
	1 (Less than monthly)	171(5)	4.23(±1.89)	1>0
	2 (Monthly)	110(3)	4.78(±2.09)	2>0, 2>1
	3 (Weekly)	63(2)	5.00(±2.16)	3>0, 3>1
	4 (Daily or almost daily)	55(2)	5.15(±2.38)	4>0, 4>1
Q9	Alcohol-related injuries			
	0 (No)	1917(60)	3.97(±1.76)	
	2 (Yes, but not in the past year)	192(6)	4.57(±2.07)	2>0
	4 (Yes, during the past year)	47(1)	4.95(±2.22)	4>0
Q10	Others concerned about your drinking			
	0 (No)	1870(58)	3.98(±1.76)	
	2 (Yes, but not in the past year)	179(6)	4.51(±2.01)	2>0
	4 (Yes, during the past year)	107(3)	4.26(±1.97)	4>0

† The detailed questionnaire of each AUDIT item was shown in Supplementary Table S1.

*Significant item categories were shown ($p<0.05$).
